# 320. Clinical Impact of Metagenomic Next Generation Sequencing in a Large Pediatric and Adult Cohort

**DOI:** 10.1093/ofid/ofac492.398

**Published:** 2022-12-15

**Authors:** Alice Lehman, Lea Goren, Olivia Toles, Shannon L Andrews, Beth K Thielen, Daniel Drozdov, Nathan Rubin

**Affiliations:** University of Minnesota, Minneapolis, Minnesota; University of Minnesota, Minneapolis, Minnesota; University of Minnesota, Minneapolis, Minnesota; University of Minnesota, Minneapolis, Minnesota; University of Minnesota, Minneapolis, Minnesota; University of Minnesota, Minneapolis, Minnesota; University of Minnesota, Minneapolis, Minnesota

## Abstract

**Background:**

Plasma metagenomic next-generation sequencing (mNGS) is an emerging diagnostic tool. As the clinical use of mNGS increases, efforts to better understand the role of mNGS in diagnosis and management of infectious diseases are essential. Current literature is limited to small retrospective reviewers and a prospective study therefore subject to institutional variability. Here, we aim to describe the use, sensitivity, time to diagnosis, clinical impact, and cost effectiveness of mNGS in the largest patient cohort to date.

**Methods:**

We included pediatric and adult patients who had plasma mNGS testing as part of care from December 2017 through December 2021 at University of Minnesota (UMN). Patients were identified using the Karius database. Electronic medical records were reviewed for every mNGS test, recording patient demographics, underlying conditions, indications, and results within 30 days of mNGS. All cases were assessed by 2 reviewers including one board-certified infectious diseases doctor. Sensitivity of mNGS was determined relative to conventional tests. The primary end point was change in clinical management. Secondary end points included accuracy, time to diagnosis, and infectious disease consultation. UMN IRB approved this study as non human subjects research.

**Results:**

584 mNGS tests were ordered in the study period, with 203 reviews completed to date. Solid organ transplantation (SOT) (32%) was the most common underlying condition, followed by hemopoietic stem cell transplantation (HSCT) (28%). Fever (45.8%), then pulmonary findings (26%) were the most common indications for ordering mNGS. Conventional and mNGS tests identified an infectious cause of the clinical syndrome in 49% of cases, while a contaminant was found in 52% of cases. The sensitivity of mNGS in identification of infectious cause was 47%, approximately 7% more sensitive than conventional testing. Despite this increased sensitivity, mNGS changed clinical management in 20% of cases.

**Conclusion:**

mNGS demonstrated superiority in accurate detection of infectious causes of clinical syndromes, however, changed clinical management in a minority of cases, suggesting timing of the mNGS test is critical. This study identified SOT and HSCT as high yield patient groups for implementation of a prospective study.

**Disclosures:**

**Beth K. Thielen, MD, PhD**, Horizon: Advisor/Consultant|Horizon: Honoraria|Merck: Grant/Research Support.

